# 21-Hy­droxy­pregna-1,4-diene-3,20-dione

**DOI:** 10.1107/S1600536811028674

**Published:** 2011-07-23

**Authors:** S. Yousuf, M. Bibi, M. I. Choudhary

**Affiliations:** a H.E.J. Research Institute of Chemistry, International Center for Chemical and Biological Sciences, University of Karachi, Karachi 75270, Pakistan

## Abstract

The title compound, C_21_H_28_O_3_, is a fungal transformed metabolite of decoxycorticosterone acetate, consisting of four fused rings *A*, *B*, *C* and *D*. Ring *A* is nearly planar, with a maximum deviation of 0.010 (3) Å from the least-squares plane, while the *trans*-fused rings *B* and *C* adopt chair conformations. The five-membered ring *D* is in an envelope conformation. The orientation of the side chain is stabilized by an intramolecular O—H⋯O hydrogen bond. In the crystal, adjecent mol­ecules are linked by C—H⋯O hydrogen bonds into extended zigzag chains along the *a* axis.

## Related literature

The title compound was previously reported as the transformed metabolite of 11-de­oxy­corticosterone, see: Holland *et al.* (1995 ▶). For the crystal structure of the closely related compound corticosterone, see: Campsteyn *et al.* (1973 ▶) and for that of of 11-de­oxy­corticosterone, see: Dideberg *et al.* (1973 ▶); Dey *et al.* (1999 ▶). 
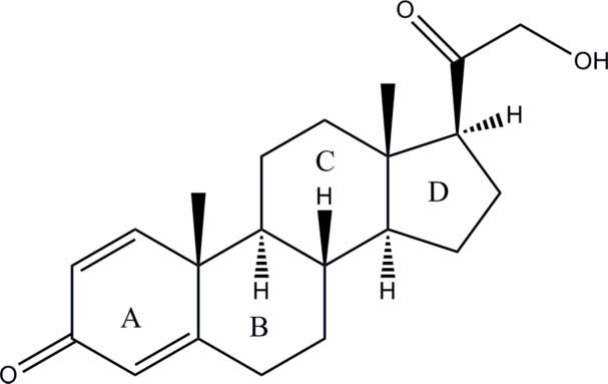


## Experimental

### 

#### Crystal data

C_21_H_28_O_3_
*M* *_r_* = 328.43
Monoclinic, 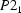

*a* = 7.5882 (9) Å
*b* = 11.3506 (13) Å
*c* = 10.5462 (12) Å
β = 102.258 (2)°
*V* = 887.64 (18) Å^3^
*Z* = 2
Mo *K*α radiation
μ = 0.08 mm^−1^
*T* = 298 K
0.36 × 0.13 × 0.12 mm

#### Data collection

Bruker SMART APEX CCD area-detector diffractometer
Absorption correction: multi-scan (*SADABS*; Bruker, 2000 ▶) *T*_min_ = 0.972, *T*_max_ = 0.990
5287 measured reflections
1739 independent reflections
1467 reflections with *I* > 2σ(*I*)
*R*_int_ = 0.026

#### Refinement

*R*[*F* ^2^ > 2σ(*F* ^2^)] = 0.038
*wR*(*F* ^2^) = 0.107
*S* = 0.95
1739 reflections
219 parameters
1 restraint
H-atom parameters constrained
Δρ_max_ = 0.14 e Å^−3^
Δρ_min_ = −0.13 e Å^−3^

### 

Data collection: *SMART* (Bruker, 2000 ▶); cell refinement: *SAINT* (Bruker, 2000 ▶); data reduction: *SAINT*; program(s) used to solve structure: *SHELXS97* (Sheldrick, 2008 ▶); program(s) used to refine structure: *SHELXL97* (Sheldrick, 2008 ▶); molecular graphics: *SHELXTL* (Sheldrick, 2008 ▶); software used to prepare material for publication: *SHELXTL* and *PLATON* (Spek, 2009 ▶).

## Supplementary Material

Crystal structure: contains datablock(s) global, I. DOI: 10.1107/S1600536811028674/zb2014sup1.cif

Structure factors: contains datablock(s) I. DOI: 10.1107/S1600536811028674/zb2014Isup2.hkl

Additional supplementary materials:  crystallographic information; 3D view; checkCIF report

## Figures and Tables

**Table 1:** Hydrogen-bond geometry (Å, °)

*D*—H⋯*A*	*D*—H	H⋯*A*	*D*⋯*A*	*D*—H⋯*A*
O2—H1*O*2⋯O3	0.91	1.98	2.602 (4)	124
C21—H22*B*⋯O1^i^	0.97	2.53	3.415 (5)	152

Symmetry code: (i) 
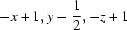
.

**Fig. 1.**
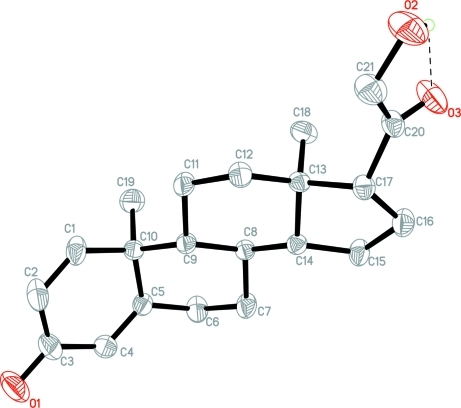
The molecular structure of (I) with displacement ellipsoids drawn at 30% probability level. The dashed lines indicates the intramolecular hydrogen bonds. Hydrogen atoms are omitted for clarity.

**Fig. 2.**
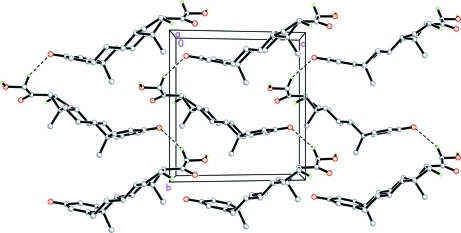
The crystal packing of the title compound I. Only hydrogen atoms involved in hydrogen bonding are shown.

## supplementary crystallographic information

### Comment

Structural modification of a substance as a result of enzymatic or metabolic activities of a living organism is known as biotransformation. In the current biotransformational study, the structural modification of a hypertension- inducing agent, 21-hydroxyprogesterone-21-acetate also known as 11-decoxycorticosterone acetate (DOCA), was investigated by using *Cunninghamella elegans* to obtain the title compound, 21-hydyoxypregna-1,4-diene-3,20-dione (I) as transformed metabolite, previously obtained as a result of the biotransformation of deoxycorticosteroid [Holland *et al.*, 1995]. The title compound posseses four fused rings A (C1–C5/C10), B (C5–C10), C (C8–C9/C11–C14) and D (C13–C17). Trans fused rings B [Q= 0.568 (3) Å, θ = 177.5 (3)° and φ = 259 (13)°]and C [Q= 0.576 (3) Å, θ = 173.4 (3)° and φ = 97 (2)°] are in chair conformations, whereas ring D adopts [Q= 0.444 (3)Å and φ = 10.6 (4)°] an envelope conformation. Ring A found Planar in geometery. The conformation of the acetyl side on C17 is stabilized by intramolecular O2–H1O2···O3 hydrogen bonding (Fig.1). In the crystal structure, the molecules are linked by C21–H22B···O1 interaction to form extended chains in a zigzag fashion (Fig. 2, Table-1). The bond dimensions are similar to those found in structurally related corticosterone [Campsteyn *et al.* 1973] and 11-deoxycorticosterone (Dey *et al.*, 1999 and Dideberg *et al.*, 1973).

### Experimental

Fungal medium was prepared by dissolving following ingredients in distilled H_2_O (4.0 *L*): glucose (40.0 g), glycerol (40.0 ml), peptone (20.0 g), potassium dihydrogen phosphate (20.0 g), and sodium chloride (20.0 g), yeast extract (20.0 g) and equally distributed in 40 conical flasks (100 ml per flask). The mouth of flasks were covered with cotton wool and autoclaved at 121 °C. The mycelia of *Cunninghamella elegans* (NRRL 1392) were transferred into flasks and incubated at 26 °C for three days on rotary shaker. After growth of *C. elegans*, 21-hydroxyprogesterone-21-acetate (1 g m, dissolved in 20 ml acetone, 0.5 ml per flask) was distributed in all the flasks and allow to grow under same conditions for 14 days, followed by the filtration and extraction with dichloromethane. The extract was dried over anhydrous sodium sulfate and evaporated to obtained brown gumy material. The gummy material was fractionated by using silica gel column chromatography (gradient petroleum ether-acetone solvent system) to obtain several fractions. The fraction obtained 25%. was finally purified by using RP-HPLC (*L*-80, methanol-water 2:1, retention time 32 min.) to obtain title compound (15 mg).

### Refinement

H atoms on methyl, methylene, methine and oxygen were positioned geometrically with C—H = 0.96 Å, 0.97 Å, 0.93 Å and O—H = 0.90 Å respectively, and constrained to ride on their parent atoms with *U*_iso_(H)= 1.2*U*_eq_(CH_2_, CH and OH) and 1.5*U*_eq_(CH_3_). The absolute configuration was assumed to be that of corticosterone (Campsteyn *et al.*, 1973) and 11-decoxycorticosterone (Diberg *et al.*, 1973) & Dey *et al.*, 1999) itself; 1538 Friedel pairs were merged.

### Crystal data

**Table:**

C_21_H_28_O_3_	*F*(000) = 356
*M**_r_* = 328.43	*D*_x_ = 1.229 Mg m^−^^3^
Monoclinic, *P*2_1_	Mo *K*α radiation, λ = 0.71073 Å
*a* = 7.5882 (9) Å	Cell parameters from 1378 reflections
*b* = 11.3506 (13) Å	θ = 2.0–25.5°
*c* = 10.5462 (12) Å	µ = 0.08 mm^−^^1^
β = 102.258 (2)°	*T* = 298 K
*V* = 887.64 (18) Å^3^	Block, colourless
*Z* = 2	0.36 × 0.13 × 0.12 mm

### Data collection

**Table:**

Bruker SMART APEX CCD area-detector diffractometer	1739 independent reflections
Radiation source: fine-focus sealed tube	1467 reflections with *I* > 2σ(*I*)
graphite	*R*_int_ = 0.026
Detector resolution: 8.33 pixels mm^-1^	θ_max_ = 25.5°, θ_min_ = 2.0°
ω scan	*h* = −9→9
Absorption correction: multi-scan (*SADABS*; Bruker, 2000)	*k* = −13→13
*T*_min_ = 0.972, *T*_max_ = 0.990	*l* = −8→12
5287 measured reflections	

### Refinement

**Table:**

Refinement on *F*^2^	Primary atom site location: structure-invariant direct methods
Least-squares matrix: full	Secondary atom site location: difference Fourier map
*R*[*F*^2^ > 2σ(*F*^2^)] = 0.038	Hydrogen site location: inferred from neighbouring sites
*wR*(*F*^2^) = 0.107	H-atom parameters constrained
*S* = 0.95	*w* = 1/[σ^2^(*F*_o_^2^) + (0.0661*P*)^2^ + 0.090*P*] where *P* = (*F*_o_^2^ + 2*F*_c_^2^)/3
1739 reflections	(Δ/σ)_max_ < 0.001
219 parameters	Δρ_max_ = 0.14 e Å^−^^3^
1 restraint	Δρ_min_ = −0.13 e Å^−^^3^

### Special details

**Table:**

Geometry. All e.s.d.'s (except the e.s.d. in the dihedral angle between two l.s. planes) are estimated using the full covariance matrix. The cell e.s.d.'s are taken into account individually in the estimation of e.s.d.'s in distances, angles and torsion angles; correlations between e.s.d.'s in cell parameters are only used when they are defined by crystal symmetry. An approximate (isotropic) treatment of cell e.s.d.'s is used for estimating e.s.d.'s involving l.s. planes.
Refinement. Refinement of *F*^2^ against ALL reflections. The weighted *R*-factor *wR* and goodness of fit *S* are based on *F*^2^, conventional *R*-factors *R* are based on *F*, with *F* set to zero for negative *F*^2^. The threshold expression of *F*^2^ > σ(*F*^2^) is used only for calculating *R*-factors(gt) *etc*. and is not relevant to the choice of reflections for refinement. *R*-factors based on *F*^2^ are statistically about twice as large as those based on *F*, and *R*- factors based on ALL data will be even larger.

### Fractional atomic coordinates and isotropic or equivalent isotropic displacement parameters (Å^2^)

**Table:**

	*x*	*y*	*z*	*U*_iso_*/*U*_eq_	
O1	0.5573 (4)	0.6466 (3)	0.9017 (2)	0.0945 (9)	
O2	0.2867 (4)	0.3644 (3)	−0.2454 (2)	0.0913 (9)	
H1O2	0.1895	0.4096	−0.2788	0.110*	
O3	0.0016 (3)	0.4543 (3)	−0.1825 (2)	0.0826 (9)	
C1	0.5718 (4)	0.6862 (3)	0.5714 (3)	0.0618 (9)	
H1A	0.6579	0.6870	0.5203	0.074*	
C2	0.6286 (4)	0.6644 (4)	0.6965 (3)	0.0685 (10)	
H2A	0.7504	0.6497	0.7289	0.082*	
C3	0.5054 (4)	0.6630 (3)	0.7843 (3)	0.0629 (8)	
C4	0.3171 (4)	0.6834 (3)	0.7260 (3)	0.0567 (8)	
H4A	0.2338	0.6820	0.7791	0.068*	
C5	0.2577 (4)	0.7042 (3)	0.5998 (3)	0.0454 (7)	
C6	0.0594 (4)	0.7164 (3)	0.5427 (3)	0.0587 (8)	
H6A	0.0351	0.7927	0.5011	0.070*	
H6B	−0.0086	0.7106	0.6107	0.070*	
C7	0.0023 (4)	0.6184 (3)	0.4432 (3)	0.0543 (8)	
H7A	0.0165	0.5428	0.4870	0.065*	
H7B	−0.1241	0.6279	0.4027	0.065*	
C8	0.1135 (3)	0.6197 (2)	0.3392 (2)	0.0374 (6)	
H8A	0.0915	0.6947	0.2924	0.045*	
C9	0.3169 (3)	0.6114 (2)	0.4003 (2)	0.0391 (6)	
H9A	0.3345	0.5360	0.4465	0.047*	
C10	0.3807 (3)	0.7096 (3)	0.5053 (2)	0.0441 (7)	
C11	0.4304 (4)	0.6053 (3)	0.2956 (3)	0.0520 (8)	
H11A	0.4262	0.6814	0.2532	0.062*	
H11B	0.5549	0.5898	0.3373	0.062*	
C12	0.3677 (3)	0.5108 (3)	0.1926 (3)	0.0496 (7)	
H12A	0.4374	0.5169	0.1257	0.059*	
H12B	0.3889	0.4335	0.2320	0.059*	
C13	0.1683 (3)	0.5243 (2)	0.1315 (2)	0.0374 (6)	
C14	0.0637 (3)	0.5206 (2)	0.2418 (2)	0.0371 (6)	
H14A	0.0989	0.4472	0.2894	0.045*	
C15	−0.1335 (4)	0.5052 (3)	0.1719 (3)	0.0515 (7)	
H15A	−0.2019	0.4657	0.2273	0.062*	
H15B	−0.1889	0.5809	0.1460	0.062*	
C16	−0.1246 (4)	0.4299 (3)	0.0534 (3)	0.0572 (8)	
H16A	−0.1928	0.4667	−0.0248	0.069*	
H16B	−0.1748	0.3524	0.0617	0.069*	
C17	0.0763 (3)	0.4196 (3)	0.0464 (2)	0.0442 (6)	
H17A	0.1233	0.3460	0.0893	0.053*	
C18	0.1330 (5)	0.6381 (3)	0.0533 (3)	0.0561 (8)	
H18A	0.1603	0.7044	0.1106	0.084*	
H18B	0.0086	0.6412	0.0094	0.084*	
H18C	0.2080	0.6403	−0.0094	0.084*	
C19	0.3717 (5)	0.8342 (3)	0.4439 (3)	0.0665 (9)	
H19A	0.3905	0.8925	0.5113	0.100*	
H19B	0.2554	0.8457	0.3881	0.100*	
H19C	0.4635	0.8415	0.3943	0.100*	
C20	0.1086 (4)	0.4173 (3)	−0.0904 (3)	0.0487 (7)	
C21	0.2832 (5)	0.3690 (4)	−0.1131 (3)	0.0672 (9)	
H22A	0.3817	0.4181	−0.0685	0.081*	
H22B	0.3013	0.2903	−0.0767	0.081*	

### Atomic displacement parameters (Å^2^)

**Table:**

	*U*^11^	*U*^22^	*U*^33^	*U*^12^	*U*^13^	*U*^23^
O1	0.104 (2)	0.128 (2)	0.0422 (12)	0.0043 (19)	−0.0030 (12)	0.0045 (16)
O2	0.1074 (19)	0.113 (2)	0.0626 (15)	0.0171 (18)	0.0394 (14)	−0.0185 (16)
O3	0.0769 (16)	0.123 (2)	0.0423 (11)	0.0197 (15)	0.0010 (11)	−0.0080 (13)
C1	0.0358 (13)	0.100 (3)	0.0503 (17)	−0.0076 (16)	0.0113 (13)	−0.0282 (18)
C2	0.0459 (16)	0.100 (3)	0.0544 (18)	0.0067 (18)	−0.0006 (15)	−0.022 (2)
C3	0.072 (2)	0.072 (2)	0.0398 (15)	−0.0014 (18)	0.0019 (15)	−0.0067 (16)
C4	0.0578 (16)	0.079 (2)	0.0381 (14)	−0.0107 (16)	0.0206 (13)	−0.0129 (15)
C5	0.0430 (13)	0.0529 (17)	0.0418 (14)	−0.0062 (12)	0.0127 (12)	−0.0123 (13)
C6	0.0418 (14)	0.086 (2)	0.0511 (17)	0.0021 (15)	0.0167 (13)	−0.0210 (17)
C7	0.0346 (13)	0.079 (2)	0.0510 (16)	−0.0073 (14)	0.0138 (12)	−0.0163 (16)
C8	0.0332 (12)	0.0424 (14)	0.0374 (12)	0.0011 (11)	0.0091 (10)	−0.0030 (11)
C9	0.0343 (12)	0.0466 (14)	0.0378 (13)	−0.0019 (11)	0.0111 (11)	−0.0039 (12)
C10	0.0390 (13)	0.0617 (18)	0.0338 (13)	−0.0067 (12)	0.0130 (11)	−0.0076 (13)
C11	0.0353 (13)	0.081 (2)	0.0430 (14)	−0.0117 (13)	0.0161 (12)	−0.0176 (15)
C12	0.0365 (14)	0.072 (2)	0.0435 (14)	0.0019 (13)	0.0164 (12)	−0.0132 (15)
C13	0.0384 (13)	0.0406 (14)	0.0347 (12)	0.0018 (11)	0.0115 (11)	−0.0028 (12)
C14	0.0350 (13)	0.0384 (13)	0.0383 (13)	−0.0001 (10)	0.0086 (11)	0.0010 (11)
C15	0.0371 (14)	0.0630 (19)	0.0541 (16)	−0.0042 (13)	0.0093 (12)	−0.0162 (15)
C16	0.0489 (15)	0.0660 (19)	0.0572 (17)	−0.0109 (15)	0.0126 (14)	−0.0192 (16)
C17	0.0481 (14)	0.0418 (14)	0.0423 (14)	−0.0010 (13)	0.0089 (12)	−0.0069 (13)
C18	0.083 (2)	0.0443 (16)	0.0436 (15)	−0.0050 (15)	0.0185 (14)	−0.0006 (13)
C19	0.085 (2)	0.061 (2)	0.0556 (19)	−0.0246 (18)	0.0177 (17)	−0.0115 (16)
C20	0.0550 (15)	0.0485 (16)	0.0416 (14)	−0.0026 (14)	0.0079 (13)	−0.0127 (13)
C21	0.075 (2)	0.074 (2)	0.0573 (19)	0.0070 (18)	0.0232 (16)	−0.0122 (17)

### Geometric parameters (Å, °)

**Table:**

O1—C3	1.231 (4)	C11—H11A	0.9700
O2—C21	1.402 (4)	C11—H11B	0.9700
O2—H1O2	0.9067	C12—C13	1.522 (4)
O3—C20	1.202 (3)	C12—H12A	0.9700
C1—C2	1.321 (4)	C12—H12B	0.9700
C1—C10	1.493 (4)	C13—C18	1.525 (4)
C1—H1A	0.9300	C13—C14	1.542 (3)
C2—C3	1.450 (5)	C13—C17	1.562 (4)
C2—H2A	0.9300	C14—C15	1.531 (3)
C3—C4	1.450 (4)	C14—H14A	0.9800
C4—C5	1.332 (4)	C15—C16	1.528 (4)
C4—H4A	0.9300	C15—H15A	0.9700
C5—C6	1.504 (4)	C15—H15B	0.9700
C5—C10	1.505 (4)	C16—C17	1.546 (4)
C6—C7	1.527 (4)	C16—H16A	0.9700
C6—H6A	0.9700	C16—H16B	0.9700
C6—H6B	0.9700	C17—C20	1.514 (4)
C7—C8	1.519 (4)	C17—H17A	0.9800
C7—H7A	0.9700	C18—H18A	0.9600
C7—H7B	0.9700	C18—H18B	0.9600
C8—C14	1.517 (3)	C18—H18C	0.9600
C8—C9	1.544 (3)	C19—H19A	0.9600
C8—H8A	0.9800	C19—H19B	0.9600
C9—C11	1.539 (3)	C19—H19C	0.9600
C9—C10	1.574 (4)	C20—C21	1.500 (4)
C9—H9A	0.9800	C21—H22A	0.9700
C10—C19	1.551 (5)	C21—H22B	0.9700
C11—C12	1.528 (4)		
			
C21—O2—H1O2	100.3	C13—C12—H12B	109.4
C2—C1—C10	125.3 (3)	C11—C12—H12B	109.4
C2—C1—H1A	117.4	H12A—C12—H12B	108.0
C10—C1—H1A	117.4	C12—C13—C18	111.0 (2)
C1—C2—C3	121.5 (3)	C12—C13—C14	107.63 (19)
C1—C2—H2A	119.2	C18—C13—C14	111.8 (2)
C3—C2—H2A	119.2	C12—C13—C17	116.7 (2)
O1—C3—C2	122.2 (3)	C18—C13—C17	109.15 (19)
O1—C3—C4	121.8 (3)	C14—C13—C17	100.0 (2)
C2—C3—C4	116.0 (2)	C8—C14—C15	119.2 (2)
C5—C4—C3	123.1 (3)	C8—C14—C13	113.4 (2)
C5—C4—H4A	118.4	C15—C14—C13	104.29 (19)
C3—C4—H4A	118.4	C8—C14—H14A	106.4
C4—C5—C6	120.9 (3)	C15—C14—H14A	106.4
C4—C5—C10	122.9 (2)	C13—C14—H14A	106.4
C6—C5—C10	116.1 (2)	C16—C15—C14	104.4 (2)
C5—C6—C7	108.8 (2)	C16—C15—H15A	110.9
C5—C6—H6A	109.9	C14—C15—H15A	110.9
C7—C6—H6A	109.9	C16—C15—H15B	110.9
C5—C6—H6B	109.9	C14—C15—H15B	110.9
C7—C6—H6B	109.9	H15A—C15—H15B	108.9
H6A—C6—H6B	108.3	C15—C16—C17	107.3 (2)
C8—C7—C6	111.6 (2)	C15—C16—H16A	110.3
C8—C7—H7A	109.3	C17—C16—H16A	110.3
C6—C7—H7A	109.3	C15—C16—H16B	110.3
C8—C7—H7B	109.3	C17—C16—H16B	110.3
C6—C7—H7B	109.3	H16A—C16—H16B	108.5
H7A—C7—H7B	108.0	C20—C17—C16	114.0 (2)
C14—C8—C7	112.6 (2)	C20—C17—C13	114.7 (2)
C14—C8—C9	108.66 (19)	C16—C17—C13	103.9 (2)
C7—C8—C9	111.0 (2)	C20—C17—H17A	108.0
C14—C8—H8A	108.1	C16—C17—H17A	108.0
C7—C8—H8A	108.1	C13—C17—H17A	108.0
C9—C8—H8A	108.1	C13—C18—H18A	109.5
C11—C9—C8	111.5 (2)	C13—C18—H18B	109.5
C11—C9—C10	113.7 (2)	H18A—C18—H18B	109.5
C8—C9—C10	112.4 (2)	C13—C18—H18C	109.5
C11—C9—H9A	106.2	H18A—C18—H18C	109.5
C8—C9—H9A	106.2	H18B—C18—H18C	109.5
C10—C9—H9A	106.2	C10—C19—H19A	109.5
C1—C10—C5	111.2 (2)	C10—C19—H19B	109.5
C1—C10—C19	108.0 (3)	H19A—C19—H19B	109.5
C5—C10—C19	109.6 (2)	C10—C19—H19C	109.5
C1—C10—C9	109.0 (2)	H19A—C19—H19C	109.5
C5—C10—C9	107.2 (2)	H19B—C19—H19C	109.5
C19—C10—C9	111.9 (2)	O3—C20—C21	117.8 (3)
C12—C11—C9	113.8 (2)	O3—C20—C17	123.1 (3)
C12—C11—H11A	108.8	C21—C20—C17	119.1 (3)
C9—C11—H11A	108.8	O2—C21—C20	112.2 (3)
C12—C11—H11B	108.8	O2—C21—H22A	109.2
C9—C11—H11B	108.8	C20—C21—H22A	109.2
H11A—C11—H11B	107.7	O2—C21—H22B	109.2
C13—C12—C11	111.2 (2)	C20—C21—H22B	109.2
C13—C12—H12A	109.4	H22A—C21—H22B	107.9
C11—C12—H12A	109.4		
			
C10—C1—C2—C3	−0.9 (6)	C10—C9—C11—C12	−179.4 (2)
C1—C2—C3—O1	−177.6 (4)	C9—C11—C12—C13	53.7 (3)
C1—C2—C3—C4	1.6 (6)	C11—C12—C13—C18	66.5 (3)
O1—C3—C4—C5	178.4 (4)	C11—C12—C13—C14	−56.2 (3)
C2—C3—C4—C5	−0.9 (5)	C11—C12—C13—C17	−167.6 (2)
C3—C4—C5—C6	175.7 (3)	C7—C8—C14—C15	53.7 (3)
C3—C4—C5—C10	−0.6 (5)	C9—C8—C14—C15	177.0 (2)
C4—C5—C6—C7	−117.8 (3)	C7—C8—C14—C13	177.0 (2)
C10—C5—C6—C7	58.7 (4)	C9—C8—C14—C13	−59.6 (3)
C5—C6—C7—C8	−56.2 (3)	C12—C13—C14—C8	61.7 (3)
C6—C7—C8—C14	178.0 (2)	C18—C13—C14—C8	−60.5 (3)
C6—C7—C8—C9	56.0 (3)	C17—C13—C14—C8	−175.93 (19)
C14—C8—C9—C11	52.3 (3)	C12—C13—C14—C15	−167.1 (2)
C7—C8—C9—C11	176.6 (2)	C18—C13—C14—C15	70.7 (3)
C14—C8—C9—C10	−178.8 (2)	C17—C13—C14—C15	−44.7 (2)
C7—C8—C9—C10	−54.4 (3)	C8—C14—C15—C16	161.2 (3)
C2—C1—C10—C5	−0.5 (5)	C13—C14—C15—C16	33.4 (3)
C2—C1—C10—C19	119.7 (4)	C14—C15—C16—C17	−8.4 (3)
C2—C1—C10—C9	−118.5 (4)	C15—C16—C17—C20	−144.8 (3)
C4—C5—C10—C1	1.3 (4)	C15—C16—C17—C13	−19.3 (3)
C6—C5—C10—C1	−175.1 (3)	C12—C13—C17—C20	−80.5 (3)
C4—C5—C10—C19	−118.0 (3)	C18—C13—C17—C20	46.4 (3)
C6—C5—C10—C19	65.5 (3)	C14—C13—C17—C20	163.8 (2)
C4—C5—C10—C9	120.4 (3)	C12—C13—C17—C16	154.4 (2)
C6—C5—C10—C9	−56.1 (3)	C18—C13—C17—C16	−78.8 (3)
C11—C9—C10—C1	−59.5 (3)	C14—C13—C17—C16	38.7 (3)
C8—C9—C10—C1	172.8 (2)	C16—C17—C20—O3	21.1 (4)
C11—C9—C10—C5	−179.9 (2)	C13—C17—C20—O3	−98.5 (3)
C8—C9—C10—C5	52.3 (3)	C16—C17—C20—C21	−160.2 (3)
C11—C9—C10—C19	59.9 (3)	C13—C17—C20—C21	80.2 (3)
C8—C9—C10—C19	−67.8 (3)	O3—C20—C21—O2	−5.9 (5)
C8—C9—C11—C12	−51.2 (3)	C17—C20—C21—O2	175.3 (3)

### Hydrogen-bond geometry (Å, °)

**Table:**

*D*—H···*A*	*D*—H	H···*A*	*D*···*A*	*D*—H···*A*
O2—H1O2···O3	0.91	1.98	2.602 (4)	124
C21—H22B···O1^i^	0.97	2.53	3.415 (5)	152

Symmetry codes: (i) −*x*+1, *y*−1/2, −*z*+1.
